# The Microstructure and Mechanical Properties of Dual-Spot Laser Welded-Brazed Ti/Al Butt Joints with Different Groove Shapes

**DOI:** 10.3390/ma13225105

**Published:** 2020-11-12

**Authors:** Peng Li, Zhenglong Lei, Xinrui Zhang, Enze Cai, Yanbin Chen

**Affiliations:** State Key Laboratory of Advanced Welding and Joining, Harbin Institute of Technology, 92 West Dazhi Street, Harbin 150001, China; hitcailiaolp@126.com (P.L.); lp19871020@126.com (X.Z.); lp20063006@163.com (E.C.); lipengddf@126.com (Y.C.)

**Keywords:** dual-spot laser: laser welding-brazing, groove shape: intermetallic compounds, tensile strength

## Abstract

Laser welding-brazing was performed to join Ti and Al together. The dual-spot laser beam mode was selected as the heat source in this study. Ti-6Al-4V and 6061-T6 Al alloys were selected as the experimental materials. Al-12Si welding wire was selected as the filler material. The effect of groove shape on the weld appearance, microstructure, temperature field, and mechanical performance of Ti/Al welded-brazed butt joints was investigated. The interfacial intermetallic compound (IMC) layer at the Ti/Weld brazing interface was inhomogeneous in joints with I-shaped and Y-shaped grooves. In Ti/Al joints with V-shaped grooves, the homogeneity of temperature field and IMC layer was improved, and the maximum thickness difference of IMC layer was only 0.20 μm. Nano-sized granular Ti_7_Al_5_Si_12_, Ti_5_Si_3_, and Ti(Al,Si)_3_ constituted the IMCs. The tensile strength of Ti/Al joints with V-shaped grooves was the highest at 187 MPa. The fracture mode transformed from brittle fractures located in the IMC layer to ductile fractures located in the Al base metal, which could be attributed to the improvement of the IMC layer at the brazing interface.

## 1. Introduction

Hybrid structures for dissimilar alloys, such as Ti/Mg [1], Fe/Al [2], and Ti/Al [3], have attracted growing attention in the aircraft and auto industries, owing to the advantages in reducing weight and saving energy. The Ti/Al joint combines the advantages of Ti (high strength and corrosion resistance) and Al (low weight and low cost) sufficiently [4]. Unfortunately, a huge challenge exists in joining Ti and Al together [5]. A brittle interfacial intermetallic compound (IMC) layer forms at the Ti/Al bonding interface [6]. The mechanical performance of Ti/Al joints is dominated by this brittle IMC layer [7]. The formation of this brittle IMC layer depends on temperature-time cycles during the welding process [8]. Some processes of joining, for example brazing [9], friction stir welding [4], arc welding-brazing [10], and laser welding-brazing [11], were studied for improving Ti/Al joint strength. Laser beams could provide an appropriate energy output and high heating/cooling rate; thus, laser welding-brazing techniques have attracted increasing attention in Ti/Al joining [12].

The characteristics of the IMC layer are critical to the tensile strength of a Ti/Al joint [13]. When the IMC layer thickness exceeded 5.0 μm, the tensile property of Ti/Al joints decreased significantly [14]. The serrated-shaped IMC layer was conducive to improving the Ti/Al joint tensile property because of the interfacial bonding area was increased [15]. A homogeneous IMC layer at the Ti/Weld brazing interface also affected the mechanical performance of Ti/Al joints [16]. To reduce the formation of brittle IMC, the laser beam position typically shifted toward the Ti side or Al side to adjust the heat distribution at the Ti/Al interface in direct laser welding-brazing of Ti/Al.

By offsetting the laser beam to the Ti side by 0.3–0.5 mm, the IMC layer was only 2.0 μm in a Ti/Al joint [8]. Through offsetting the laser beam to the Ti side by 0.75 mm, a sound Ti/Al butt joint was obtained, and the tensile strength reached 191 MPa [17]. In addition, in most studies regarding laser welding-brazing Ti/Al, the laser beam was offset to the Al side to expand the process window [18]. The IMC layer thickness in laser welded-brazed Ti/Al joints decreased with the increase of the laser offset distance toward the Al side [19]. Compared to laser beams focused on the Ti side, the thickness of the IMC layer decreased from 190–300 μm to 20 μm, when the laser beam shifted to the Al side by 0.2 mm [20]. However, the wetting ability of molten metal was poor, and the interfacial metallurgical reaction was hardly controlled in the laser welding-brazing of Ti/Al without a filling material [21]. These problems could be solved with a filling material.

Through filling with an Al-12Si wire, a laser welded-brazed Ti/Al joint with 278 MPa was obtained [22]. Through modulating the traditional circular spot to a rectangular spot, the homogeneity of the IMC layer and the tensile property of a laser Ti/Al joint were all improved [16]. The position of the laser beam was also usually focused on the Al side to inhibit the intermetallic reaction during the laser joining of Ti/Al with a filling material. The proper offset distance was 0.3 mm from the centerline toward the Al side in laser welding-brazing Ti/Al with a filling of AlMg4.5MnZr wire [23].

In addition to laser welding-brazing, laser-arc hybrid welding was also employed in Ti/Al joining. Due to the stable welding process and the lower heat input, laser-CMT (cold metal transfer) was employed for joining Ti/Al, and the thickness of the IMC layer was only 1.0 μm [13]. A Ti/Al joint with 230 MPa was obtained by laser-MIG (metal-inert gas welding) hybrid welding-brazing, and the homogeneity of the IMC was improved by modifying the heat source offset and laser-deviation angle [24].

In the previous introduction, the mode of the laser beam was traditional single-spot. Compared to single-spot, dual-spot laser beams were shown to increase the quality of the weld surface, and were adopted in joining similar metals [25]. By selecting the dual-spot laser mode, the porosity defects in laser welded Mg joints decreased [26]. During the dual-spot laser welding process, the stability and area of the weld pool were improved [27]. For dissimilar metal joining, the dual-spot laser mode also improved the weld quality. In dual-spot laser welding of steel/Al lap joints, the wettability of the filler material on the surface of steel was improved, due to the preheating induced by the leading laser spot in tandem dual-spot [28]. By employing the dual-spot laser mode, the wetting length of the filler metal in steel/Al lap joint was increased from 3.0 mm to 4.2 mm [29]. The formation of pore defects in the Fe/Al lap joint was reduced by CW (continuous wave mode)/PW (plus wave mode) dual-spot laser welding-brazing [30]. For Fe/Al joints in the coach peel configuration, the tensile strength of the joint obtained by adopting a parallel deal-spot laser was 20 MPa higher than that of tandem dual-spot laser, as the IMC thickness was also reduced [31]. The studies regarding dual-spot laser welding-brazing dissimilar metal butt joints were limited. The porosity defects and spreadability of the filler metal in Fe/Al butt joints were improved through adopting the dual-spot laser mode [32]. The temperature field along the brazing interface was more uniform in parallel dual-spot Fe/Al joints than with tandem dual-spot [32]. A double half-spot tandem spot was adopted for joining Ti/Al, and the homogeneity of the IMC layer along the Ti/Weld brazing interface and the tensile strength of the joint were all improved [12].

Except for the type of heat source, the shape of the brazing interface in dissimilar joints also affected the temperature field, thereby affecting the joint performance [33]. For laser welded-brazed Ti/Al joints with an I-shape groove for the Ti side, the distribution of the IMC layer along the brazing interface was inhomogeneous. The IMC layer thickness was approximately 5.1 μm in the middle region and about 2.2 μm in the top region [23]. A Y-shaped groove shape for the Ti side was adopted in the TIG Ti/Al welding process to improve the spreadability of the filler material; however, the IMC layer was unevenly distributed at the brazing interface in the thickness direction [34]. The temperature gradient along the brazing interface was decreased during laser welding-brazing Ti/Al butt joints with V-shaped grooves for the Ti side, and this improved the tensile properties of the joint [16]. For single-spot laser welding-brazing of Fe/Al, the smallest temperature gradient (172 °C) was obtained under a half-V groove shape, while the temperature gradient was 380 °C and 278 °C in joints with square-shape and half Y grooves, respectively [35]. Research found that the temperature field at brazing was affected by the groove shape. Compared to a 30° bevel angle, the 45° V-shape groove on the Ti side was beneficial for the flow of molten wire during dual-spot laser welding-brazing of Ti/Al [12]. However, the studies regarding the influence of groove shape on the microstructure and mechanical performance of dual-spot laser welded-brazed Ti/Al butt joints were limited.

In the present paper, Ti/Al butt joints with different groove shapes were obtained through laser welding-brazing under dual-spot mode. The microstructure of the interfacial IMC layer was studied using scanning electron microscopy (SEM) and transmission electron microscopy (TEM). The tensile properties of Ti/Al joints were tested, and the fractured surface was investigated using SEM. The interfacial temperature field in joints with different groove shapes was calculated. Finally, the relationship between the temperature field, IMC layer, and tensile behavior was clarified. The purpose of this paper was to confirm the proper groove shape for dual-spot laser welding-brazing Ti/Al.

## 2. Materials and Methods 

We chose 2.0 mm thick Al alloy (6061-T6) and Ti alloy (Ti-6Al-4V) as base materials (BM) in this experiment. The dimensions of the Ti and Al samples were machined into 50 mm (width) × 100 mm (length) for joining. Al-Si based welding wire was previously selected as the filler metal in joining Ti/Al [16] or Fe/Al [32] because the Si element could suppress the interfacial reaction. Thus, in our study, Al-12Si flux-cored welding wire containing 12.0 wt% Si was chosen as the filler metal. The diameter of the welding wire was 1.2 mm. The detailed chemical contents of the BM and wire are listed in Table 1. Noncorrosive Nocolok (KAlF_4_ 65 wt% and K_3_AlF_6_ 35 wt%) was used to improve the spreadability of the filler metal. The temperature field at the brazing interface was related to the groove shape of the brazing interface [12,16,23,34]. Therefore, three typical groove shapes (I-shaped, Y-shaped, and V-shaped) for the Ti side were prepared in this study. The groove shapes at the Al side were contained as V-shaped with 45°. To confirm the influence of groove shape, the welding parameters of the Ti/Al joints in this study were consistent and are displayed in Table 2.

**Figure 1:**
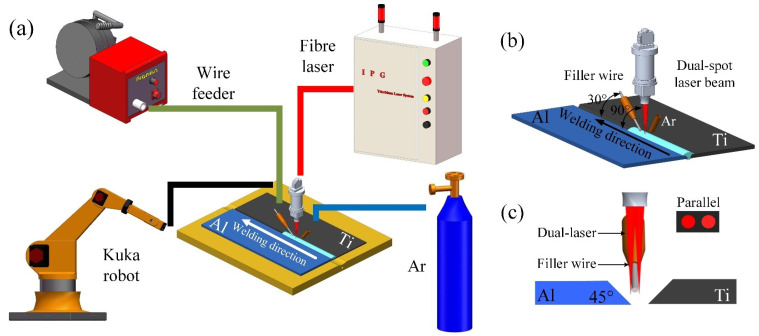
(**a**,**b**) schematic figures of the welding process; (**c**) a diagram of a parallel dual-spot laser beam.

**Figure 2:**
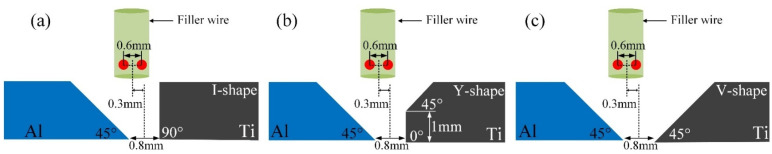
The schematics of three groove shapes: (**a**) I-shaped; (**b**) Y-shaped; (**c**) V-shaped.

**Figure 3:**
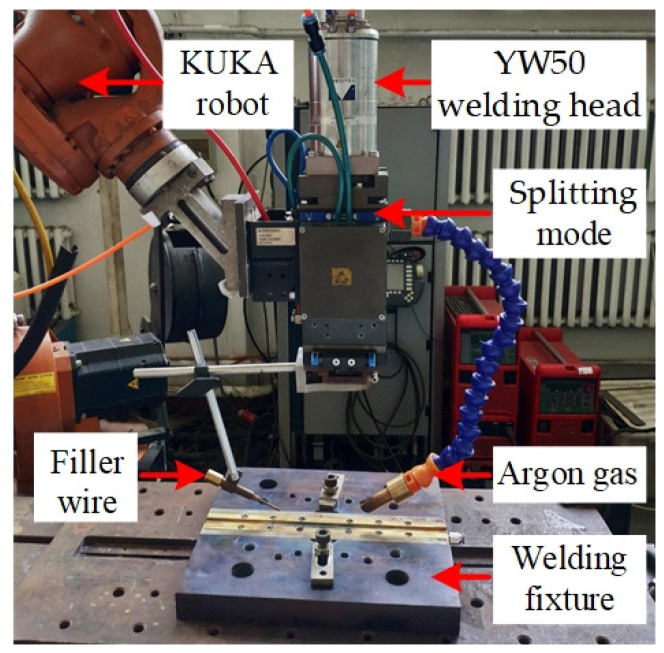
The actual experimental set-up.

The set-up of the Ti/Al dual-spot laser welding-brazing system is shown in Figure 1. The laser system was integrated with a fiber laser (IPG YLR-5000, Oxford, USA) and a six-axis robot (Kuka KR16-2, Augsburg, Germany). The wavelength of the laser beam was 1070 nm. The maximum output laser power was 5.0 kW. Through a splitting mode in the Precitec YW50 welding head (Gaggenau, Germany), the dual-spot laser was obtained through dividing the single spot laser. The arrangement of the dual-spots was selected as a parallel mode to increase the weld pool area. The distance of the parallel dual spots of the laser was 0.6 mm. A diagrammatic sketch of the parallel dual spots in this study is shown in Figure 1c. To avoid laser melted Ti BM, the laser beam was focused on the Al side. The offset distance of the laser spot was 0.3 mm from the center-line of the weld toward the Al side. The details regarding the dual-spot laser welding-brazing and groove shapes are shown in Figure 2. The actual experimental set-up is shown in Figure 3.

Before welding, the BM were soaked in acetone and ground using abrasive paper for removing the oil and oxide film on the surface. The Nocolok flux powder was dissolved in acetone and formed the flux suspension. Before welding, the prepared flux suspension was spread homogenously on the groove and the surface of Ti BM for improving the wettability of the liquid metal. The thickness of the coating was controlled at about 0.2 mm. Argon gas was used as to avoid the oxidation of the weld. After welding, the cross-section of Ti/Al joint was prepared perpendicularly to the welding direction by a wire electrical discharging machine.

The cross-section specimens were first ground with abrasive paper (#200, #400, #600, #800, and #1000), then polished with diamond polishing pastes (3.0 and 1.0 μm), and finally a mirror-polished cross-section specimen was obtained. The microstructure of the IMC was examined using SEM (FEI Helios NanoLab 600i, Hillsboro, OR, USA). The contents of the IMC were confirmed using energy dispersive X-ray spectroscopy (EDS). The electron voltage was 20 kV, the beam current was 0.69 nA, the work distance was 6.0 mm during EDS testing. The IMC layer thickness was analyzed using ImagePro software. The phase indentation IMC layer was performed using TEM (FEI Talos F200x, Hillsboro, OR, USA).

Tensile tests were conducted to evaluate the mechanical properties of the Ti/Al joints with different groove shapes. The tensile tests were performed on an AG-X Plus universal testing machine with a loading rate of 1.0 mm × min^−1^ under room temperature. The size of the testing samples was prepared following the ISO 6892: 1998 international standard. For each Ti/Al joint, three testing samples were tested to guarantee the accuracy of the tensile properties of the joints. After the tensile tests, the fracture surface at the Al side of fractured Ti/Al joints was inspected using SEM.

## 3. Results and Discussion

### 3.1. Surface and Cross-Sections Characteristics in Ti/Al Welds

**Figure 4:**
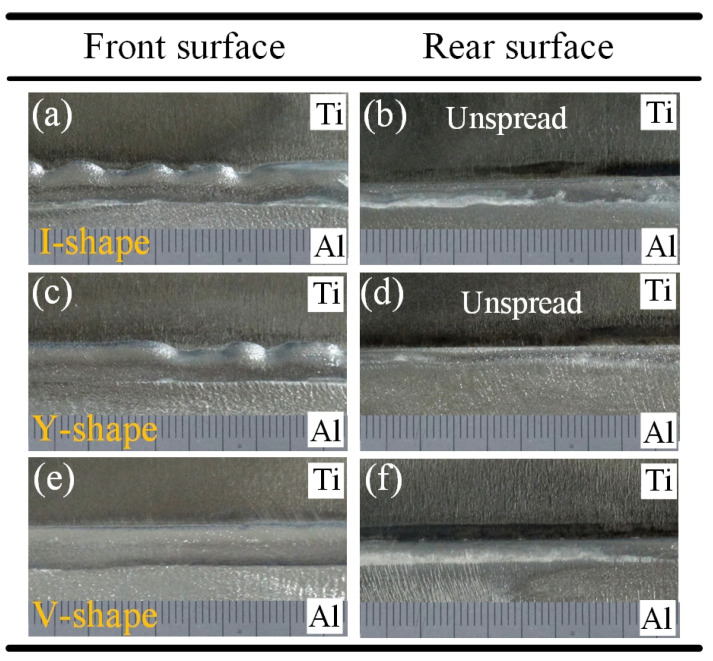
The weld face appearance of joints: (**a**,**b**) I-shaped; (**c**,**d**) Y-shaped; (**e**,**f**) V-shaped.

**Figure 5:**
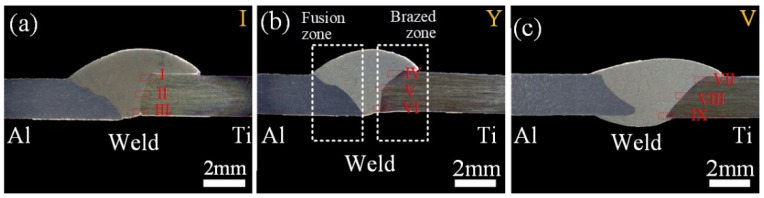
The cross-sections of joints:(**a**) I-shaped; (**b**) Y-shaped; (**c**) V-shaped.

The appearance of the Ti/Al weld seam was determined using the spreadability of molten Al-12Si filler wire on the surface of the Ti alloy. The weld face appearance and cross-sections of joints with different groove shapes are shown in Figure 4 and Figure 5. The front surface of a joint with an I-shaped groove was discontinuous; however, the filler metal was fully wetted on the surface of Ti as shown in Figure 4a. However, the weld seam was narrow in the rear surface of the Ti/Al joint, and the filler metal was not wetted on the surface for the Ti side as shown in Figure 5b. As displayed in cross-section in Figure 5a, the filler metal was fully spread on the whole brazing interface; however, the wetting length of the filler material on the rear surface for the Ti side was very small.

A similar result was also observed in Fe/Al joints with square shaped grooves [36]. The weld face appearance of joints with Y-shaped grooves was similar to those with I-shaped grooves. The front surface was discontinuous, and the rear weld seam was narrow as shown in Figure 4c,d, The filler material did not successfully wet on the rear surface for the Ti side as shown in Figure 5b. For V-shaped grooves, the front weld face appearance of the Ti/Al joint became smooth and continuous in Figure 4e. The width of the weld seam on the rear surface was increased in Figure 4f. The filler material was successfully wetted on the rear surface for the Ti side as shown in Figure 5c.

These results indicate that the spreadability of the filler metal on the rear surface of Ti was related to the groove shape for the Ti side. As mentioned in [37], the increase of wetting temperature was beneficial to improve the spreadability of the filler metal. According to the result of simulation of the temperature field, the peak temperatures were 717 °C and 739 °C in the bottom region of joints with I-shaped and Y-shaped grooves. However, the peak temperature was 750 °C in the bottom region of joints with V-shaped grooves. This increase in temperature was responsible for the improvement of the filler material on the rear surface of Ti with a V-shaped groove, which was clearly the highest of the three groove shape conditions.

The V-shaped groove was beneficial to obtain a sound Ti/Al joint. The fusion zone and brazed zone formed a complete Ti/Al joint. The metlted Al and filler material formed the fusion zone. The unmelted Ti and filler metal formed the brazed zone. For the three joints, the Ti/weld interface remained linear as shown in Figure 4. The results indicated that Ti remained solid in the whole welding-brazing process and that the production of excessive brittle IMC could be prevented. This indicated that the weld parameters selected in this study were appropriate.

### 3.2. Effect of Groove Shape on the Interfacial Structure

**Figure 6:**
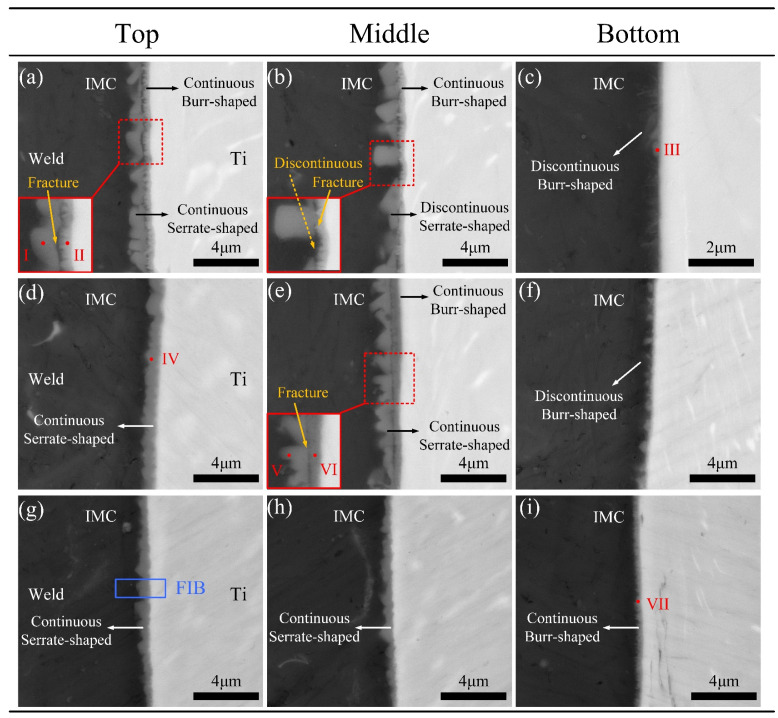
Interfacial intermetallic compound (IMC) microstructures in Ti/Al joints with different groove shapes: (**a**–**c**) I-shaped; (**d**–**f**) Y-shaped; (**g**–**i**) V-shaped.

**Figure 7:**
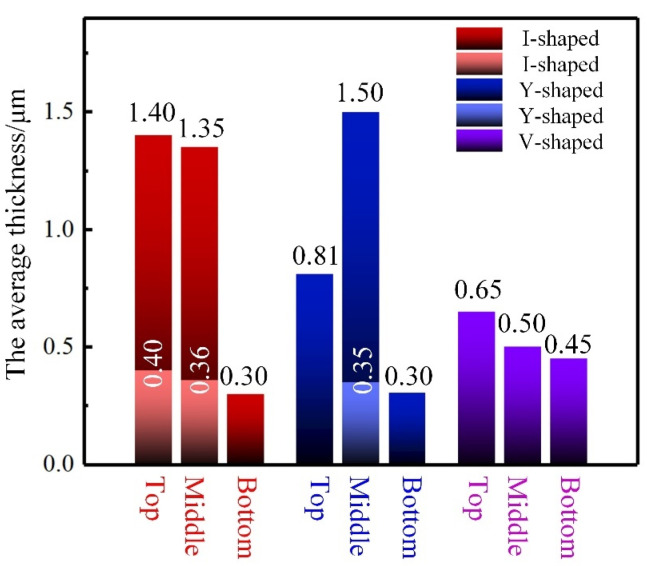
The average thickness of the IMC layers in joints with I-shaped, Y-shaped, and V-shaped grooves.

The IMC layer in the brazed zone dominated the mechanical performance of the Ti/Al joint. Figure 6 shows the typical morphology of the IMC layer from top to bottom along the brazing interface in joints, marked as I–IX in Figure 5. The average thickness of the IMC layer is given in Figure 7. We noticed that the morphology of the IMC modified significantly with the change of groove shape for the Ti side.

For joint with an I-shaped groove, a mixed IMC layer was found in the top region as shown in Figure 6a. The mixed IMC layer was composed of two parts: a 0.40-μm-thick continuous burr-shaped IMC close to the Ti BM and a 1.00-μm-thick continuous serrate-shaped IMC close to the weld. The magnified image of red dotted rectangular region is shown in the lower left corner in Figure 6a. A fracture existed in the interface between these two IMC layers. We inferred that the formed IMC layer grew non-homogeneously during the laser welding process in the top region of the joint with an I-shaped groove. This phenomenon also occurred in the middle region in Figure 6b. However, the serrated-shaped IMC became discontinuous in the middle region, as shown in the lower left corner in Figure 6b. A 0.30-μm-thick discontinuous burr-shaped IMC layer was observed in the bottom region in Figure 6c. The discontinuous IMC layer indicates that Ti and the weld did not form an effective metallurgical bond in the bottom region. This inhomogeneous IMC layer reduced the tensile property of the joint.

Compared to the joint with I-shaped grooves, the IMC layer transformed from a mixed IMC layer to a 0.81-μm-thick continuous serrated-shape in the top region at the brazing interface in joint with a Y-shaped groove. The inhomogeneous IMC layer in the middle region was composed of a 1.15-μm-thick continuous serrated-shaped IMC and a 0.35-μm-thick continuous burr-shaped IMC in Figure 6e. The IMC layer was discontinuous and burr-shaped in the bottom region, and only 0.30 μm thick.

In joint with a V-shaped groove, the IMC layer presented a continuous serrated-shape in the top and middle regions, and the thicknesses were 0.65 μm and 0.50 μm, respectively. As mentioned in [15], the serrated-shaped IMC was useful for improving the brazing interface bonding strength, due to its larger bonding area. In the bottom region, a 0.45-μm-thick continuous burr-shaped IMC layer was found as shown in Figure 6i. The IMC layer at the brazing interface in the thickness direction of joints with V-shaped grooves was the most homogeneous, and the thickness difference was only 0.20 μm.

To analyze the contents of IMCs with different shapes, EDS analysis was performed in positions marked as I–VII as shown in Figure 6. The positions (I, II, V, and VI) were chosen for EDS analysis to clarify the chemical compositions of the mixed IMC layer. The EDS results in Table 3 indicated that the chemical compositions of IMCs with different shapes were all similar to that of TiAl_3_ (Al 75 at%, Ti 25 at%). Due to similar crystal structures, Si could substitute Al atoms in TiAl_3_. This TiAl_3_ that contained Si atoms was typically written Ti(Al,Si)_3_ in previous work [38]. As mentioned in [39], a Ti_7_Al_5_Si_12_ phase formed at the brazing interface in Ti/Al joints with the filling of Al-12Si; the formation of this phase could thin the IMC layer. However, similar results were hardly observed through SEM in this study as shown in Figure 6. To further analyze the action of Si in interfacial reactions at the brazing interface, TEM analyses were conducted. Based on the tensile test results, the joint with a V-shaped groove that had the highest tensile strength was chosen for the TEM analysis.

**Figure 8:**
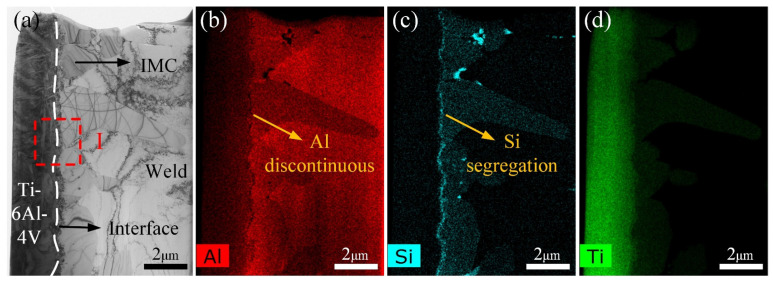
(**a**) the bright-field (BF) transmission electron microscopy (TEM) image of a brazing interface. The black phase is the Ti BM. The greyish phase is the weld. The dark grey serrated-shaped phase between the Ti and weld is the IMC, as marked by black arrows. The Ti/IMC interface is marked as a white dotted line; (**b**–**d**) the corresponding Al, Si, and Ti element distribution maps.

Focused ion beam (FIB) cutting technology was employed for preparing the TEM samples and the FIB position is marked in Figure 6g. Figure 8 shows the bright-field (BF) TEM image of interfacial microstructures and the corresponding Al, Si, and Ti element distribution maps. As shown in Figure 8a, three kinds of phases were observed. The black phase is the Ti BM. The greyish phase is the weld. The dark grey serrated-shaped phase between the Ti and weld is the IMC, as marked by black arrows in Figure 8a. The Ti/IMC interface is marked as a white dotted line in Figure 8a. A thin layer composed of granular phases was observed at the Ti/IMC interface in Figure 8a.

This phenomenon is more clearly observed in elemental distribution maps as shown in Figure 8b–d. The distribution of the Al element along the Ti/IMC interface is discontinuous in Figure 8b. The Al element was missing in some regions along Ti/IMC interface. In contrast, the Si element is clearly segregated at the Ti/IMC interface in Figure 8c. The element absence or segregation of the Ti element along the Ti/IMC interface is not observed in Figure 8d. These results indicated that other Ti-Si or Ti-Al-Si phases might form at the Ti/IMC interface. However, the size of granular phases was too small for electron diffraction. Therefore, the high-resolution transmission electron microscopy (HRTEM) and corresponding fast Fourier transform (FFT) technology were chosen for analyzing these granular phases.

**Figure 9:**
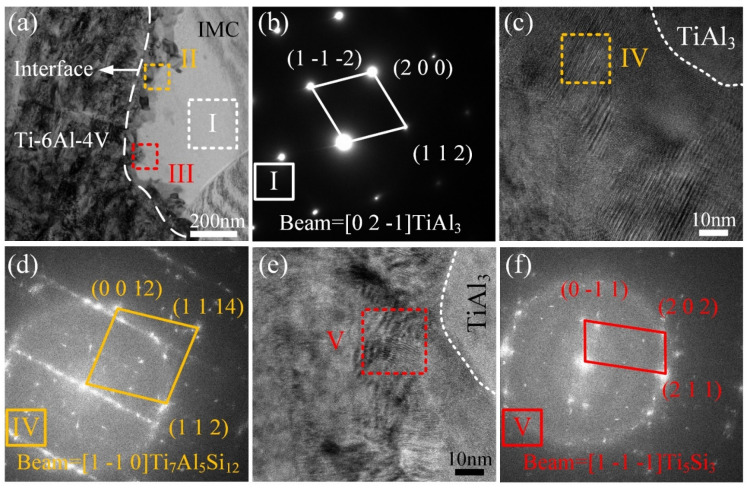
(**a**) magnified BF TEM image of region I in Figure 8a; (**b**) corresponding SAED pattern of I phase; (**c**,**e**) high-resolution transmission electron microscopy (HRTEM) images of regions II and III in (**a**); (**d**,**f**) corresponding fast Fourier transform (FFT) of the IV and V phases.

Figure 9a is the magnified image of region I in Figure 8a. Nanosized phases were found at the Ti/IMC interface. The contrast of these nanosized granular phases was different, as marked by II and III in Figure 9a. The SAED (selected area electron diffraction) pattern of region I in Figure 9a also indicated that the I phase is TiAl_3_. Figure 9c shows the HRTEM image of region II in Figure 9a. We determined that the crystal plane spacing of the IV phase and TiAl_3_ was different. The FFT of region IV given in Figure 9d confirms that the type of IV phase is Ti_7_Al_5_Si_12_. The HRTEM image of region III in Figure 9a is shown in Figure 9e. Another phase with different crystal plane spacing with TiAl_3_ as found and marked as V in Figure 9e. The corresponding FFT also indicated that this phase is Ti_5_Si_3_. Nanosized Ti_7_Al_5_Si_12_ and Ti_5_Si_3_ phases were found at the Ti/IMC interface; these granular phases led to the uneven distribution of Al and Si at the Ti/IMC interface. The formation process of the Ti_7_Al_5_Si_12_ and Ti_5_Si_3_ phases consumed Ti atoms diffusing from the Ti BM, and the formation of the brittle TiAl_3_ phase was inhibited, which reduced the thickness of the IMC layer. This was beneficial for improving the mechanical performance of the joints.

### 3.3. Thermal History

**Figure 10:**
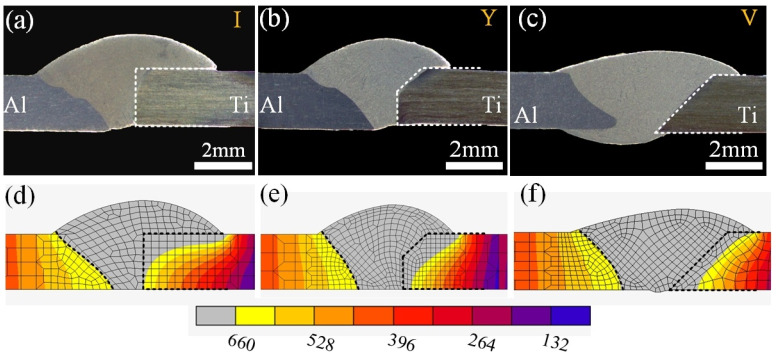
The verification of experimental cross-sections and numerical models of joints: (**a**,**d**) I-shaped; (**b**,**e**) Y-shaped; (**c**,**f**) V-shaped.

Except for the alloying element, the formation process of the IMC layer was dominated by the thermal history at the brazing interface. Therefore, the thermal history of joints with different groove shapes was calculated using the FEM (Finite Element Method) software MSC. Figure 10 shows the comparison of experimental cross-sections and the numerical results. For ensuring the convergence of the calculation process, the weld reinforcement of joint with V-shaped groove was removed, this would not affect the accuracy of the calculated result. The difference between experimental fusion lines and numerical ones was small in Figure 10, and this indicated that the adopted numerical model in this study was reasonable.

**Figure 11:**
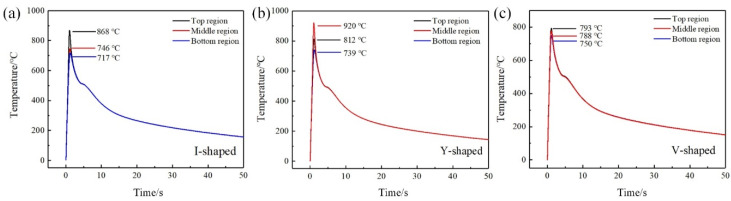
The thermal cycles at the brazing interface of joints: (**a**) I-shaped; (**b**) Y-shaped; (**c**) V-shaped.

Figure 11 shows the temperature cycle in the top, middle, and bottom regions at the brazing interface in joints with I-shaped, Y-shaped, and V-shaped grooves. For joints with I-shaped and V-shaped grooves, the peak temperature in the top region was the highest, and was the lowest in the bottom region. However, the highest peak temperature was in the middle region in joints with Y-shaped grooves. The values of peak temperature and the temperature gradients of joints with the three groove shapes were all different. For joints with I-shaped grooves, the peak temperatures from top to bottom were 868, 746, and 717 °C in Figure 11a. The temperature gradient was 151 °C. For joints with Y-shaped grooves, the peak temperatures were 812, 920, and 739 °C from top to bottom, and the temperature gradient was 181 °C, which was the maximum in the three joints. The highest peak temperature in the middle region was 920 °C, and this result was consistent with the IMC distribution in Figure 6. The IMC layer in joints with Y-shaped grooves were the thickest in the middle region, which was 1.50 μm. For joints with V-shaped grooves, the peak temperatures were 793, 788, and 750 °C from top to bottom. The temperature gradient was 43 °C, which was the smallest in the three joints. This result was consistent with the IMC layer distribution. The V-shaped groove for the Ti side improved the homogeneity of the temperature field at the brazing interface; thus, the distribution of the IMC layer was the most homogeneous.

### 3.4. Mechanical Performance

**Figure 12:**
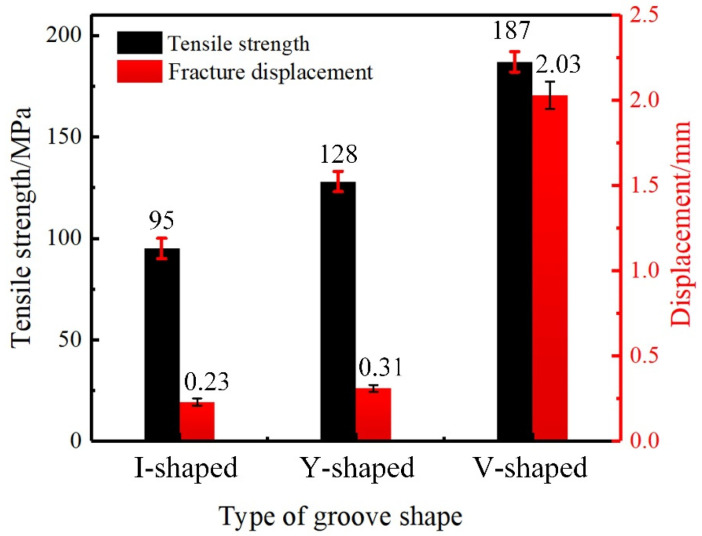
The tensile strength–displacement curves of Ti/Al joints with different groove shapes.

**Figure 13:**
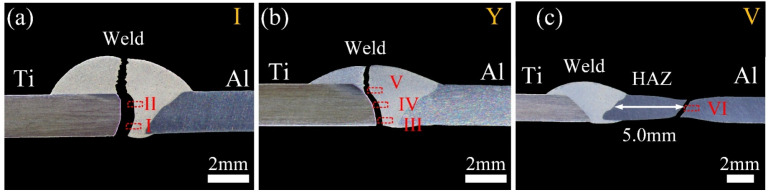
The cross-sections of fractured Ti/Al joints: (**a**) I-shaped; (**b**) Y-shaped; (**c**) V-shaped.

**Figure 14:**
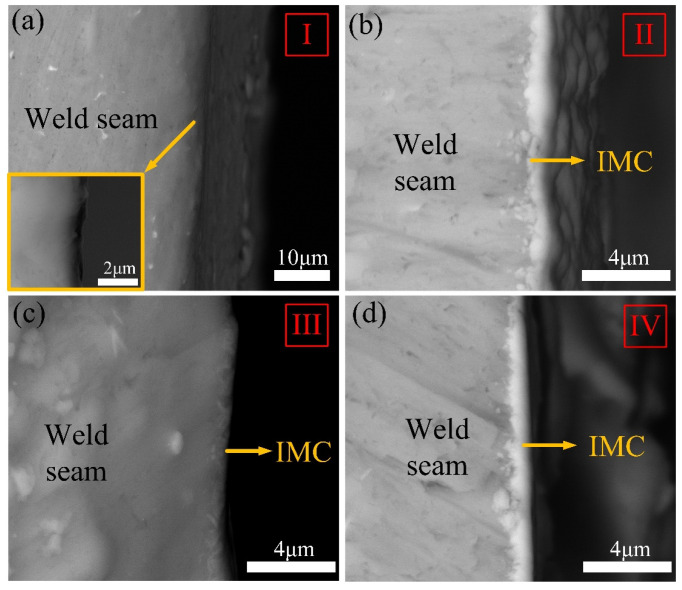
Magnified images of the fractured interface: (**a**) region I in Figure 13a; (**b**) region II in Figure 13a; (**c**) region III in Figure 13b; (**d**) region IV in Figure 13b.

**Figure 15:**
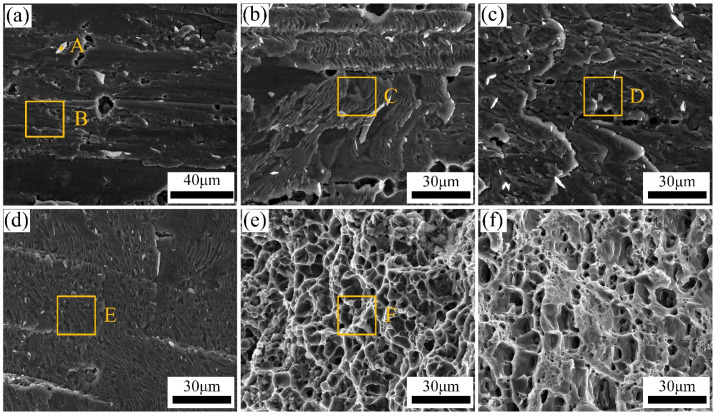
Fractured surface of Ti/Al joints: (**a**,**b**) I-shaped; (**c**–**e**) Y-shaped; (**f**) V-shaped.

The tensile test was chosen for assessing the influence of the groove shape on the mechanical performance of Ti/Al joints. The tensile strength of Ti/Al joints with different shaped grooves is given in Figure 12. The results suggested that the groove shape had a remarkable influence on the tensile properties of Ti/Al joints. The tensile strength of joints with I-shaped grooves was only 95 MPa, and the fractured displacement was only 0.23 mm. The tensile strength and fractured displacement of joints with Y-shaped grooves increased to 128 MPa and 0.31 mm. When the groove shape was V-shaped, the tensile strength and fractured displacement reached up to 187 MPa and 2.03 mm, respectively. The homogeneous IMC layer and larger interfacial bonding area in joints with V-shaped grooves were responsible for the increase of the tensile properties. To further study the influence of the groove shape on the tensile behavior of joints, the cross-sections and fractured surface of joints were both examined, and the results are displayed in Figure 13, Figure 14 and Figure 15. The magnified SEM image of the fractured interface that occurred at the brazing interface is shown in Figure 14 to analyze the relationship between the crack and the IMC.

Figure 13 displays the cross-sections of fractured Ti/Al joints with different groove shapes. The fractured location was closely correlated to the groove shape. The fractured position of joints with I-shaped grooves developed along the brazing interface, and almost no weld metal was left on the Ti side as shown in Figure 13a. Regions I and II in Figure 13a were selected for SEM analysis, and the magnified image of the fractured interface is shown in Figure 14a,b. The fractured interface was straight, and no IMC was found in the fractured interface in the bottom region, as shown in the magnified image in the left corner of Figure 14a. The corresponding fractured surface was a flat surface in Figure 15a. However, particles marked by A were found in the fractured surface. The particle phase was Ti(Al,Si)_3_, and the matrix phase was Al-Si filler metal, based on the content in Table 4. This was due to the ineffective metallurgical combination shown in Figure 6c, as the filler material in the bottom region directly peeled off from the brazing interface and formed a flat fractured surface. In region II, a clear IMC layer was found in the magnified image of the fracture interface as shown in Figure 14b. The corresponding fractured surface is a typical cleavage plane as shown in Figure 15b. The content of region C is the Ti(Al,Si)_3_ phase. As revealed in [40], TiAl_3_ is an extremely brittle phase and a crack generation source during loading. The fracture mode of Ti/Al joints with I-shaped grooves was typical brittle fractures. The discontinuous IMC layer in the bottom region and inhomogeneous IMC layer in the other regions caused this fractured surface morphology.

With the groove shape changed to Y-shape, the fracture path of the Ti/Al joints was changed. The fracture path of joints developed along the brazing interface in the middle and bottom regions but transformed to the weld in the top region in Figure 13b. This change increased the joint tensile property. Regions III, IV, and V from the bottom to the top of the fractured joint were analyzed using SEM. For region III, a burr-shaped IMC layer was found in the fractured interface as marked by the white arrow in Figure 14c. For region IV, an obvious IMC layer was found in the fractured interface in Figure 14d. The corresponding fractured surface was a typical cleavage surface in Figure 15c,d.

The phase in the fractured surface in the middle and bottle regions was Ti(Al,Si)_3_. The inhomogeneous IMC layer in the bottom and middle regions was fractured during tensile testing. However, the characteristic of the fractured surface in the top region was dimples in Figure 15d. The content in Table 4 confirmed that this fracture was composed of Al-Si filler metal. The fractured position in the top region of joints transformed from IMC to filler metal, due to the continuous serrated-shaped IMC layer as shown in Figure 6d. Therefore, a mixed fracture of plastic and brittle mode occurred in joints with Y-shaped grooves. Compared to joints with I-shaped grooves, more weld metal was left on the Ti side in joints with Y-shaped grooves after the tensile test. The tensile strength enlarged from 95 MPa to 128 MPa.

As the groove shape changed to V-shaped, fractures of joints developed in the heat-affected zone at the Al side as shown in Figure 13c. The tensile strength was the highest among the three joints, at 187 MPa. The fractured surface was composed of dimples, which was a typical plastic fracture, as shown in Figure 15e. The maximum tensile strength of joints was lower than that of Al BM (320 MPa). This was due to the over-aging induced by the welding heat input that occurred in the heat-affected zone of Al BM. The strengthening phases in HAZ (Heat Affected Zone) were coarsened and dissolved into the 6061-T6 Al matrix.

**Figure 16:**
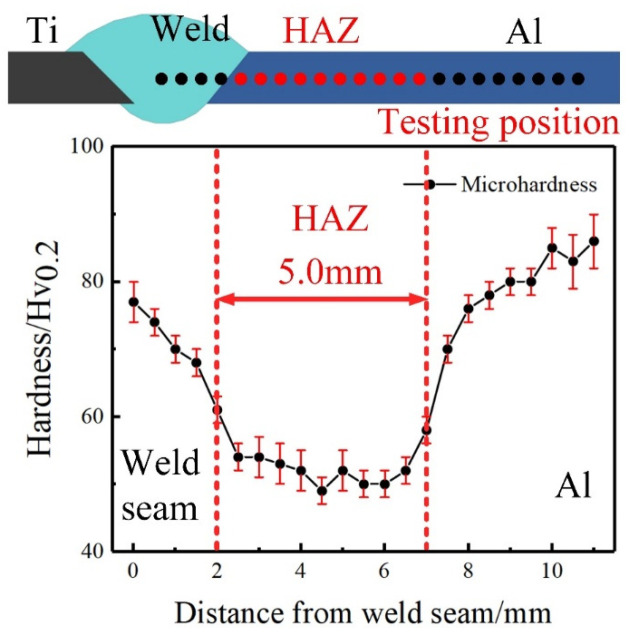
Microhardness profiles from fusion line to Al BM in joints with V-shaped grooves.

The microhardness profile of joints with V-shaped grooves was measured to confirm the existence of HAZ. Vickers microhardness measurements were conducted on the cross-sections by adopting a load of 200 g for a dwell time of 15 S. The microhardness values were measured every 0.5 mm from the weld to the Al BM. Figure 16 shows the microhardness distribution of joints with V-shaped grooves. The microhardness value of the weld was 70.0 Hv, and that of 6061-T6 Al BM was 81.1 Hv. The microhardness in HAZ was significantly decreased, and was only 52.4 Hv. The width of the softened zone reached up to 5.0 mm. This softened zone led to a decrease in the tensile strength of the Al matrix in HAZ at the Al side and, thereby, decreased the tensile properties of the Ti/Al joint.

Based on the above results of the IMC layer and the tensile behavior, we inferred that the groove shape affected the IMC layer and, furthermore, affected the tensile strength of the Ti/Al joints. For Ti/Al joints with I-shaped grooves, the inhomogeneous mixed IMC in the top and middle regions and the discontinuous burr-shaped IMC in the bottom region caused the brittle fractures during the tensile test. When the groove was Y-shaped, the 0.81-μm-thick serrated-shaped IMC formed in the top region of the joint caused the fracture to be transformed to filler metal. This resulted in an increase of the tensile strength of the joint. However, the inhomogeneous mixed IMC in the middle region and discontinuous burr-shaped IMC in the bottom region decreased the joint tensile property. When the groove shape was a V-shaped groove, a homogeneous 0.53 ± 0.12-μm-thick serrated-shaped IMC along the whole Ti/Weld brazing interface enhanced the tensile properties.

## 4. Conclusions

We successfully employed the dual-spot laser mode in laser joining Ti and Al with filling the Al-12Si wire. The effect of groove shape for the Ti side on the temperature field, the interfacial IMC, and the tensile behavior of the joint was analyzed. Our conclusions can be summarized:

(1) When the groove shape was I-shaped and Y-shaped, the weld face appearance was discontinuous, and the rear surface of the Ti alloy was not successfully wetted. With the groove shape changed to V-shape, the Ti/Al joint was sound with a satisfactory weld face appearance.
(2) The characteristics of the IMC layer were affected by the groove shapes for the Ti side. An inhomogeneous IMC layer was generated at the brazing interface in joints with I-shaped and Y-shaped grooves. For joints with V-shaped grooves, the IMC layer was the most homogeneous, and the thickness difference was only 0.20 μm. The phases of the IMC were Ti(Al,Si)_3_ phase and nanosized Ti_7_Al_5_Si_12_ and Ti_5_Si_3_ phases.
(3) The groove shape for the Ti side affected the temperature field at the brazing interface, and then affected the formation process of the IMC layer. The smallest temperature gradient was obtained in joints with V-shaped grooves, which was 43 °C, resulting in the most homogeneous IMC layer. The uneven temperature field in joints with I-shaped and Y-shaped grooves resulted in an inhomogeneous IMC layer.
(4) The tensile strength of the Ti/Al joint was dominated by the morphology and distribution of the IMC layer. An inhomogeneous IMC layer decreased the tensile property of joints with I-shaped and Y-shaped grooves. The continuous serrated-shaped IMC layer enhanced the interfacial bonding strength of joints with V-shaped grooves. The highest tensile strength reached 187 MPa.

## Figures and Tables

**Table 1:** The chemical compositions of 6061-T6 Al, Ti6Al4V, and Al-12Si (wt%).

Material	Al	Si	Fe	Cu	Zn	Mn	Mg	Ti	V
6061-T6 Al	Bal.	0.63	0.29	0.27	0.01	0.07	1.00	0.02	-
Ti6Al4V	6.21	-	0.135	-	-	-	-	Bal.	3.93
Al-12Si	Bal.	12.0	0.80	0.30	0.20	0.05	0.10	-	-

**Table 2:** The welding parameters.

Welding Parameters	Value
Laser power/W	2200
Welding speed/(m × min^−1^)	0.36
Wire feed speed/(cm × min^−1^)	288
Defocus/mm	+20
Laser offset from weld centerline/mm	0.3
Flowrate of shielding gas (Argon)/(L × min^−1^)	15

**Table 3:** The contents of regions I–VII in IMC in Figure 6 (at%).

Test Zone	Element		
	Ti	Al	Si
I	23.4	68.0	8.6
II	21.8	61.6	15.6
III	24.2	68.8	7.0
IV	25.6	66.3	8.1
V	25.6	64.6	9.8
VI	20.4	64.3	15.3
VII	24.6	65.3	10.1

**Table 4:** The contents of regions I–VI in Figure 14 (at%).

Test Zone	Element		
	Ti	Al	Si
A	21.7	67.0	11.4
B	1.6	91.8	6.6
C	23.1	68.5	8.4
D	23.4	64.2	12.4
E	25.7	66.8	7.5
F	0.0	92.0	8.0
